# Alternative folding to a monomer or homopolymer is a common feature of the type 1 pilus subunit FimA from enteroinvasive bacteria

**DOI:** 10.1074/jbc.RA119.008610

**Published:** 2019-05-24

**Authors:** Dawid S. Żyła, Andrea E. Prota, Guido Capitani, Rudi Glockshuber

**Affiliations:** From the ‡ Institute of Molecular Biology and Biophysics, ETH Zurich, Otto-Stern-Weg 5, CH-8093 Zurich and; the § Laboratory of Biomolecular Research, Division of Biology and Chemistry, Paul Scherrer Institute, Forschungsstrasse 111, CH-5232 Villigen PSI, Switzerland

**Keywords:** protein folding, X-ray crystallography, Escherichia coli (E. coli), protein structure, protein stability, alternative folding, chaperone-usher pili, FimA, type 1 pilus, urinary tract infections

## Abstract

Adhesive type 1 pili from enteroinvasive, Gram-negative bacteria mediate attachment to host cells. Up to 3000 copies of the main pilus subunit, FimA, assemble into the filamentous, helical quaternary structure of the pilus rod via a mechanism termed donor-strand complementation, in which the N-terminal extension of each subunit, the donor strand, is inserted into the incomplete immunoglobulin-like fold of the preceding FimA subunit. For FimA from *Escherichia coli*, it has been previously shown that the protein can also adopt a monomeric, self-complemented conformation in which the donor strand is inserted intramolecularly in the opposite orientation relative to that observed for FimA polymers. Notably, soluble FimA monomers can act as apoptosis inhibitors in epithelial cells after uptake of type 1-piliated pathogens. Here, we show that the FimA orthologues from *Escherichia coli*, *Shigella flexneri*, and *Salmonella enterica* can all fold to form self-complemented monomers. We solved X-ray structures of all three FimA monomers at 0.89–1.69 Å resolutions, revealing identical, intramolecular donor-strand complementation mechanisms. Our results also showed that the pseudo-palindromic sequences of the donor strands in all FimA proteins permit their alternative folding possibilities. All FimA monomers proved to be 50–60 kJ/mol less stable against unfolding than their pilus rod-like counterparts (which exhibited very high energy barriers of unfolding and refolding). We conclude that the ability of FimA to adopt an alternative, monomeric state with anti-apoptotic activity is a general feature of FimA proteins of type 1-piliated bacteria.

## Introduction

**Figure 1.**
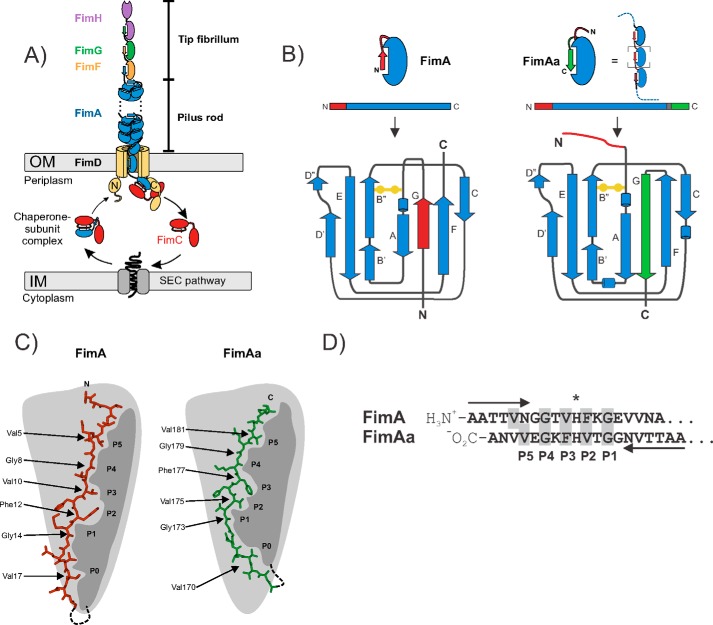
**Schematic representation of the alternative folding possibilities observed for *E. coli* FimA.** *A,* architecture and subunit composition of type 1 pili from *E. coli*. The type 1 pilus is composed of a linear tip fibrillum formed by the adhesin FimH plus the minor subunits FimG and FimF, and the helical pilus rod is formed by up to 3000 subunits of the main pilus subunit FimA. The pilus is anchored to the bacterial outer membrane by the assembly platform FimD, which catalyzes pilus assembly from the periplasmic chaperone-subunit complexes. *B,* topology diagram of the immunoglobulin-like β-sheet fold of FimA monomers in which the N-terminal donor strand is inserted intramolecularly in a parallel orientation relative to the C-terminal F-strand (*left*) and of FimAa that bears a second copy of the donor strand at its C terminus (*right*). FimAa could potentially either insert the N-terminal (*red*) or the C-terminal (*green*) donor strand into its fold, but only the more stable conformer in which the C-terminal donor strand is inserted in the antiparallel orientation relative to the F-strand is formed. This conformer corresponds to the intermolecular donor strand complementation mechanism observed between neighboring subunits in the pilus rod. *C,* schematic showing the donor strand side chains accommodated by the respective pockets (P0–P5) of the FimA IG-fold, for both FimA and FimAa. *D,* the pseudo-palindromic sequence of the FimA donor strand with its center at His^11^ (*) that allows donor strand insertion in opposite orientations.

Numerous enteroinvasive, Gram-negative pathogens bear filamentous type 1 pili on their surface that mediate bacterial attachment and pathogen internalization by epithelial cells (1–7). Type 1 pili from uropathogenic *Escherichia coli* strains represent the best-studied pilus system. These supramolecular, extracellular protein complexes recognize terminal mannoses on glycoprotein receptors of uroepithelial membranes via the adhesin FimH, the terminal pilus subunit at the distal end of the pilus (8, 9). FimH, together with the minor subunits FimF and FimG, forms a flexible tip fibrillum that is connected to the distal end of the pilus rod (Fig. 1*A*) (10). The rod contains between several hundred and 3000 copies of the main structural pilus subunit, FimA, which polymerizes to an ∼72–Å wide, right-handed helical quaternary structure with 3.13 subunits per turn and an axial rise of 7.8 Å per subunit (11, 12). The FimA subunits of the pilus rod are single domain proteins of 16 kDa with an incomplete, immunoglobulin (IG)^2^-like fold that lacks the C-terminal β-strand (G-strand). In the structure of the pilus rod, the FimA subunits interact via inter-molecular donor strand complementation, in which the N-terminal extension of each subunit, termed the donor strand, inserts as a β-strand into the preceding subunit and completes its IG-like fold (Fig. 1*B*) (11, 13, 14). The pilus rod is anchored to the outer bacterial membrane via the assembly platform FimD, which catalyzes pilus assembly and mediates subunit translocation through the membrane (15, 16) (Fig. 1*A*). All pilus subunits show intrinsically slow folding rates that would represent a kinetic bottleneck for pilus assembly *in vivo* if subunit folding was not catalyzed in the periplasm by the chaperone FimC (14, 17–20) (Fig. 1*A*). In addition, as only FimC-subunit complexes are assembly competent and recognized by FimD, FimC represents a kinetic assembly trap that prevents premature subunit assembly in the periplasm (20).

In 2010, Sukumaran *et al.* (21) discovered that the FimA proteins from the enteroinvasive pathogens *E. coli*, *Shigella flexneri*, and *Salmonella enterica* have a second function besides formation of homopolymeric pilus rods; soluble forms of FimA, independent of FimC, act as inhibitors of host cell apoptosis after pathogen internalization by stabilizing the interaction between hexokinase and the voltage-dependent anion channel (VDAC) on the surface of mitochondria. In the following work, we show that the FimA orthologues from all three pathogens can indeed adopt alternative, assembly incompetent, monomeric conformations that likely represent the anti-apoptotic FimA form (13, 21). The previously solved NMR structure of the *E. coli* FimA monomer showed that it is capable of Intramolecular self-complementation, in which the N-terminal donor strand is inserted in the opposite orientation relative to that observed for inter-molecular donor strand complementation in FimA polymers (parallel to the C-terminal F-strand of FimA) (Fig. 1*B*) (13, 14). Further analysis indicated that the origin of these alternative folding possibilities of FimA lies in the pseudo-palindromic sequence of the FimA donor strand, with its center at His^11^ and two glycines (Gly^8^ and Gly^14^) three residues to either side (Fig. 1, *C* and *D*) (13). Comparison of the NMR structure of the FimA monomer with that of a FimA variant, FimAa (which bears a second donor strand copy at its C terminus that inserts in the pilus rod-like, antiparallel orientation into the FimA-fold), revealed that the five donor strand side chain-binding pockets (P1–P5) of FimA were occupied by similar residues in both FimA conformers, with Gly^8^ and Gly^14^ defining the register of donor strand insertion (13, 14). Although FimAa could have incorporated either the N- or C-terminal donor strand into its fold, it exclusively folded to the conformer with the C-terminal donor strand inserted (antiparallel to the FimA F-strand). In contrast to FimA, FimAa proved to be extraordinarily stable against unfolding by denaturants and showed stability comparable with that of the pilus rod (14, 17). FimAa can, therefore, be considered a monomeric model for studying the structure and stability of FimA in the context of the pilus rod.

The fact that the pseudo-palindromic element of the FimA donor strand is conserved among the FimA proteins of enteroinvasive bacteria (13) and the observation that soluble forms of FimA from pathogenic *E. coli*, *S. flexneri*, and *S. enterica* strains all exhibited anti-apoptotic activity in cultured epithelium cells (21) raised the question of whether the ability to fold to two distinct conformations with different functions is a general property of FimA proteins from Gram-negative pathogens. In this study, we addressed this question by testing the FimA proteins (FimA^ECO^, FimA^SHI^, and FimA^SAL^) and the respective FimAa variants (FimAa^ECO^, FimAa^SHI^, and FimAa^SAL^) from all three pathogens for their ability to adopt the two alternative conformations that previously had only been detected for *E. coli* FimA.

## Results

### FimA^ECO^, FimA^SHI^, and FimA^SAL^ fold to self-complemented monomers via intramolecular, parallel donor strand complementation

**Figure 2.**
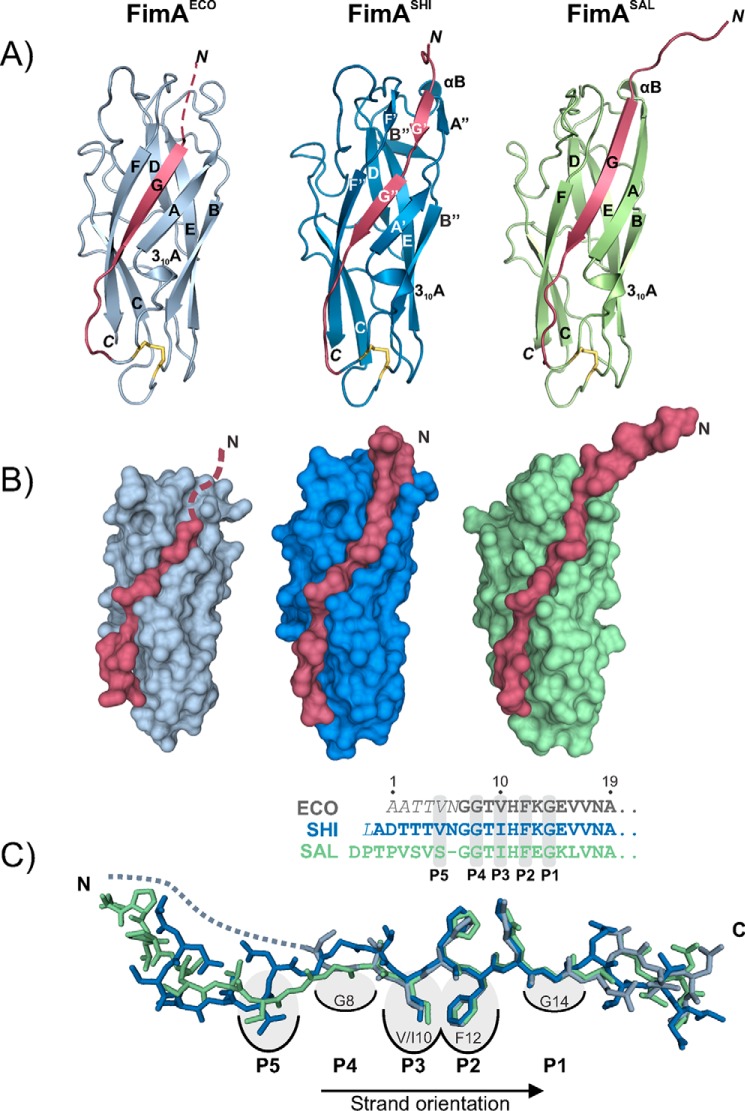
**X-ray structures of the self-complemented FimA monomers.** *A,* cartoon representations of FimA^ECO^ (*gray*), FimA^SHI^ (*blue*), and FimA^SAL^ (*green*) X-ray structures, with the N-terminal donor strand highlighted in *red. B,* surface representation of the three FimA monomer structures, highlighting the surface complementarity between the donor strands (*red*) and the FimA folds. *C,* global superposition of the donor strand conformations in all structures. In contrast to the structures of FimA^ECO^ and FimA^SAL^, the residues occupying the pockets P4 and P5 are separated by two residues in the FimA^SHI^ structure. The sequences of all three donor strands are indicated. The unresolved region of FimA^ECO^ donor strand is shown as a *dotted line*.

FimA^ECO^, FimA^SHI^, and FimA^SAL^ were produced as reduced, insoluble proteins in the *E. coli* cytoplasm without their N-terminal signal sequences. All proteins were refolded in the absence of the chaperone FimC under oxidizing conditions to allow the formation of the single, invariant structural disulfide bond. The purified proteins were crystallized, and their X-ray structures were determined at 1.5, 0.89, and 1.69 Å resolution, respectively (Fig. 2, Table 1). All FimA orthologues crystallized as self-complemented monomers, and all structures strongly resembled the previously reported NMR structure of the FimA^ECO^ monomer (13). The Cα RMSD between the most representative model of the self-complemented FimA^ECO^ NMR structure (2M5G, model 10, calculated with OLDERADO server (22)) and FimA^ECO^ X-ray structure was 0.84 Å. Specifically, all three FimA monomers showed nearly identical immunoglobulin-like folds, completed by intramolecular donor strand insertion in a parallel orientation relative to the C-terminal F-strand (Fig. 2). In contrast to the intermolecular donor strand complementation (antiparallel to the FimA F-strand) observed for the assembled *E. coli* pilus rod, where the FimA side chains of Gly^8^, Val^10^, Phe^12^, Gly^14^, and Val^16^ from the donor strand occupy the binding pockets P1–P5 of the FimA-fold, the reversed (parallel) donor strand orientation in X-ray structure of self-complemented FimA^ECO^ showed the side chain occupancies P1/Gly^14^, P2/Phe^12^, P3/Val^10^, and P4/Gly^8^ (amino acid numbering according to *E. coli* FimA) (11). Due to a lack of electron density, the occupancy of P5 with Val^16^, previously identified in the NMR structure of FimA^ECO^, could not be confirmed in the X-ray structure, but the two structures otherwise proved to be essentially identical. The global folds and registers of parallel donor strand insertion in the X-ray structures of FimA^SHI^ and FimA^SAL^ were the same as those observed in FimA^ECO^, and all three structures showed high surface complementarity between donor strand and the rest of the folded FimA domain (Fig. 1, *A* and *B*). The overall pairwise Cα RMSD values for the three X-ray structures were 0.30 Å for FimA^ECO^/FimA^SHI^ (113 Cα atoms), 0.51 Å for FimA^ECO^/FimA^SAL^ (154 Cα atoms), and 0.57 Å for FimA^SAL^/FimA^SHI^ (110 Cα atoms), and the only minor conformational differences were restricted to loop segments 15–20, 37–45, and 89–97, and residues 51 and 81 (Fig. S1). The only significant difference in the mode of donor strand insertion was found for FimA^SAL^, where Ser^6^ instead of Val^5^ occupied the P5 pocket, which caused a more extended conformation of the FimA^SAL^ donor strand segment Ser^6^–Gly^7^–Gly^8^ (Fig. 2*C*). Overall, the X-ray structures of FimA^ECO^, FimA^SHI^, and FimA^SAL^ demonstrated that the ability of FimA orthologues to adopt a self-complemented, monomeric conformation is based on the pseudo-palindromic nature of their donor strand, and that the register of donor strand insertion is dictated by the two invariant glycines (Gly^8^ and Gly^14^) in the donor strand, which are the only residues that can be accommodated by the very shallow pockets P1 and P4 without disrupting the β-sheet hydrogen-bonding network between the donor strand and the neighboring strand A and F. The fact that the pseudo-palindromic donor strand element Gly–hydrophilic–hydrophobic–Xaa–hydrophobic–hydrophilic–Gly is conserved in FimA subunits of type 1 piliated, enteroinvasive pathogens (13) predicts that most FimA orthologues share the ability to either polymerize to pilus rods or fold to monomers.

**Table 1:** **Statistics of X-ray structure determination of the FimA monomers from *E. coli, S. flexneri*, and *S. enterica***

Protein crystal	FimA *E. coli*	FimA *S. flexneri*	FimA *S. enterica*
**PDB code**	5NKT	5LP9	6ERJ
**Data collection**			
Space group	H 3 2	P 1 2_1_ 1	C 2 2 2_1_
Cell dimensions			
*a*, *b*, *c* (Å)	87.0, 87.0, 162.7	24.1, 53.8, 50.3	35.0, 104.4, 182.9
α, β, γ (°)	90, 90, 120	90, 100.3, 90	90, 90, 90
Resolution (Å)	29.87–1.50 (1.55–1.50)*^a^*	26.89–0.89 (0.92–0.89)	45.72–1.69 (1.73–1.69)
*R*_meas_ (%)	5.5 (160.2)	4.4 (56.7)	12.2 (216.9)
*I*/σ(*I*)	20.0 (1.1)	20.9 (2.5)	12.1 (1.3)
Anisotropy	0.437	0.122	0.276
CC_1/2_	99.9 (47.2)	99.9 (80.6)	99.9 (43.9)
Redundancy	9.4 (5.9)	6.2 (4.0)	15.2 (11.0)
Completeness (%)	98.8 (89.3)	91.0 (84.3)	98.6 (81.5)
Resolution *I*/σ(*I*) >2 (Å)	1.58	0.89	1.78
**Refinement**			
Resolution (Å)	29.87–1.50	26.89–0.89	45.72–1.69
No. unique reflections	37,771	88,551	37,847
*R*_work_, *R*_free_ (%)	15.8, 18.8	11.0, 12.6	17.2, 23.7
No. atoms	1,237	1,679	2,474
Protein	1,059	1,446	2,241
Ligand	25		12
Water	153	233	221
Average *B*-factors (Å^2^)	44.3	14.9	39.5
Protein	43.4	13.3	38.5
Ligand	56.1		58.6
Water	48.5	24.7	48.5
Wilson *B*-factor (Å^2^)	26.2	8.3	28.7
RMSD			
Bond lengths (Å)	0.004	0.007	0.009
Bond angles (°)	0.66	1.05	0.85
Ramachandran statistics			
Favored regions (%)	97.96	94.30	97.46
Allowed regions (%)	2.04	5.70	2.54
Outliers (%)	0	0	0

*^a^* Values in parentheses are for the highest-resolution shell.

### Folding and stability of FimA^ECO^, FimA^SHI^, and FimA^SAL^ compared with their FimAa counterparts

We next compared the thermodynamic stability of the monomeric FimA orthologues FimA^ECO^, FimA^SHI^, and FimA^SAL^ with the stability of the respective FimAa variants (Fig. 1), in which a hexa-glycine linker followed by a second copy of the donor strand was fused to the C terminus of FimA. Theoretically, the FimAa constructs can either incorporate the N- or C-terminal copy of their donor strand in the parallel or antiparallel orientation, respectively. In a previous study, we determined the NMR structure of *E. coli* FimAa (FimAa^ECO^), which showed that FimAa^ECO^ exclusively folds to the more stable, pilus rod-like conformer in which the C-terminal donor strand is inserted in the antiparallel orientation, whereas the N-terminal donor strand was not incorporated into the fold and stayed flexibly disordered. In addition, the FimAa conformer can be readily distinguished from FimA due to its dramatically increased stability against unfolding (14). We purified FimAa^ECO^, FimAa^SHI^, and FimAa^SAL^ after oxidative refolding *in vitro* from insoluble aggregates. Like FimAa^ECO^, FimAa^SHI^ and FimAa^SAL^ only adopted the more stable conformer in which the C-terminal donor strand copy was incorporated into the FimA-fold (see below).

**Figure 3.**
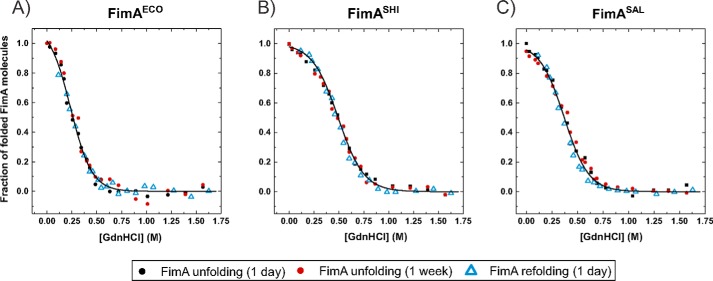
**GdnHCl-dependent equilibrium unfolding transitions at pH 7.0 and 25 °C of FimA monomers: *A,* FimA^ECO^; *B,* FimA^SHI^ ; and *C,* FimA^SAL^.** Unfolding and refolding transitions were followed via the change in the far-UV CD signal at 230 nm. Unfolding was recorded after incubation for 1 day (*black circles*) and 1 week (*red circles*), and refolding after incubation for 1 day (*blue triangles*). Identical protein concentrations of 10 μm were used in all experiments. Transitions were normalized and fitted globally to the two-state model of folding (*solid lines*) for each FimA variant. The deduced free energies of folding and cooperativities (equilibrium *m*-values) are listed in Table 2.

**Figure 4.**
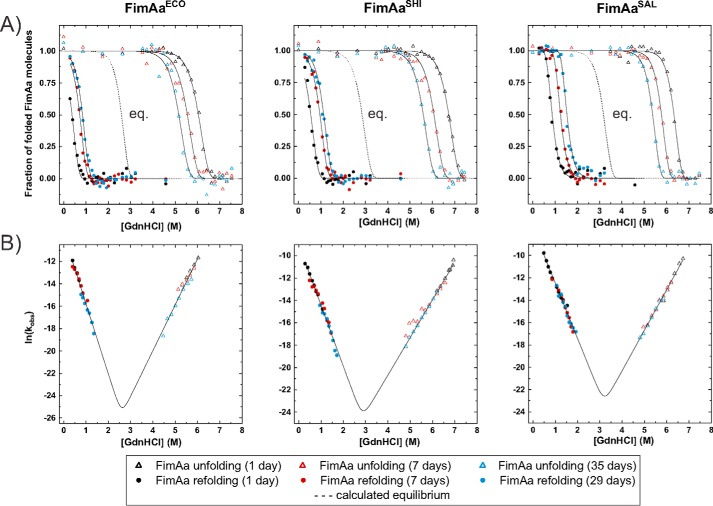
**Nonequilibrium unfolding and refolding transitions at pH 7.0 and 25 °C of FimAa variants.** *A,* unfolding and refolding transitions (*triangles* and *circles*, respectively) were recorded via the far-UV CD signal at 230 nm after incubation for 1 day (*black*), 7 days (*red*), and 35 days (*blue*). The final protein concentration was 10 μm in all experiments. The three data sets for unfolding and the three datasets for refolding recorded for each FimAa variant were fitted globally (*solid lines*) according to an unattained two-state equilibrium model and normalized (see “Materials and methods”). The equilibrium transitions calculated according to Equation 6 are indicated with *dotted lines. B,* V-plots of the logarithm of the observed rate constant of folding/unfolding (*k*_obs_) *versus* GdnHCl concentration for all FimAa constructs. Data points from the transition regions in *A* where the fraction of folded molecules was in the range of 0.05–0.95 were converted to first-order rate constants. Data were fitted according to the two-state model of folding (Equation 5, *solid lines*). The deduced values of Δ*G*^0^, the rate constants of folding and unfolding in the absence of denaturant (*k_F_*^H2O^ and *k_U_*^H2O^), and the kinetic *m*-values (*m_U_* and *m_F_*) are listed in Table 2.

Fig. 3 shows the guanidine hydrochloride (GdnHCl)-dependent unfolding equilibria of the self-complemented WT monomers FimA^ECO^, FimA^SHI^, and FimA^SAL^ at pH 7.0 and 25 °C. All proteins unfolded/refolded reversibly and attained their folding equilibria after 1 day of incubation. Evaluation of the data according to the two-state model of folding revealed that all FimA orthologues proved to be only marginally stable, with free energies of folding at zero denaturant (Δ*G*^0^) of only −5.5, −8.9, and −7.2 kJ/mol for FimA^ECO^, FimA^SHI^, and FimA^SAL^, respectively (Fig. 3, Table 2). In striking contrast, none of the FimAa orthologues reached its folding equilibrium under these conditions, even after prolonged incubation (Fig. 4*A*). Instead, the transitions of all FimAa constructs were characterized by unfolding at high and refolding at low denaturant concentrations. Although the unfolding and refolding transitions moved toward each other with increasing incubation time, they stayed widely separated even after 1 month of incubation (Fig. 4*A*). The nonequilibrium transitions of the FimAa orthologues proved to be fully consistent with an unattained two-state equilibrium in which the native and unfolded states are separated by a huge activation energy barrier (17, 23). Specifically, global analysis of all unfolding and refolding transitions of the FimAa variants recorded after different incubation times according to two-state folding yielded the characteristic, V-shaped plots of two-state folders in which the logarithm of the observed rate constant of folding/unfolding is plotted against denaturant concentration (Fig. 4*B*). Although the folding/unfolding equilibria could not be attained, this analysis allowed the calculation of the free energy of folding (Δ*G*^0^), the rate constants of folding and unfolding in the absence of denaturant (*k_F_* ^H2O^ and *k_U_*^H2O^) and the kinetic *m*-values of folding and unfolding (*m_F_* and *m_U_*) (Fig. 4, Table 2). The results showed that FimAa^ECO^, FimAa^SHI^, and FimAa^SAL^ are highly stable proteins with almost identical Δ*G*^0^ values of −66.5, −62.4, and −67.7 kJ/mol, respectively. This 7–12–fold increase in stability compared with the respective FimA counterpart is in full agreement with the extreme resistance of type 1 pili against dissociation and unfolding (17, 24). In addition, the extrapolated *k_U_* ^H2O^ values of all three FimAa orthologues were in the range of 10^−16^ s^−1^ (Table 1), indicating that kinetic resistance against unfolding is the main source of their high thermodynamic stability. All FimAa orthologues exhibited calculated incubation times of 300–3000 years to reach folding equilibrium at 25 °C. Moreover, their practically identical kinetic α-values ((*m*F)/(*m_U_* − *m_F_*)) of ∼0.6 (Table 2) indicate that the accessible surface area (ASA) of their transition state of folding is more similar to the ASA of the native state than that of the unfolded state (25).

**Table 2:** **Thermodynamic and kinetic folding parameters at pH 7.0 and 25 °C of the FimA and FimAa orthologues from *E. coli, S. flexneri,* and *S. enterica***

	FimA^ECO^	FimA^SHI^	FimA^SAL^	FimAa^ECO^	FimAa^SHI^	FimAa^SAL^
**Δ***G*^0^(kJ mol^−1^)	−5.5 ± 0.5	− 8.9 ± 0.5	−7.2 ± 2.0	−66.5 ± 2.8	−62.4 ± 1.4	−67.7 ± 1.4
*m*_eq_ (kJ mol^−1^ m^−1^)	23.0 ± 1.3	18.5 ± 1.0	18.8 ± 2.5	25.7 ± 0.9	21.9 ± 0.4	21.3 ± 0.4
***D*_1/2, eq_** (m GdnHCl)*^a^*	0.24	0.49	0.38	2.59	2.85	3.18
*t*_eq_*^b^*	<1 day	<1 day	<1 day	3225 years	978 years	273 years
**Δ***G*^0^(**Aa**)/**Δ***G*^0^(**Awt**)				12.0	7.0	9.4
*k_F_*^H2O^ (s^−1^)				7.5 ± 1.9 × 10^−5^	1.3 ± 0.2 × 10^−4^	5.5 ± 0.8 × 10^−4^
*m_F_* (m^−1^)				−6.3 ± 0.3	−5.5 ± 0.1	−5.0 ± 0.1
*k_U_* ^H2O^ (s^−1^)				1.6 ± 1.8 × 10^−16^	1.5 ± 0.8 × 10^−16^	7.5 ± 4.1 × 10^−16^
*m_U_* (m^−1^)				4.1 ± 0.2	3.4 ± 0.1	3.6 ± 0.1
*D*_1/2, kin_(m GdnHCl)*^c^*				2.6 ± 0.1	2.9 ± 0.1	3.2 ± 0.1
α = (*m_F_*/(*m_U_* − *m_F_*))*^d^*				0.60 ± 0.02	0.62 ± 0.01	0.58 ± 0.01

*^a^* The midpoint of transition determined from equilibrium experiments (*D*_1/2_ = Δ*G*^0^/*m*_eq_) (Fig. 3).

*^b^* Calculated time to reach the GdnHCl-dependent folding equilibrium (2% CD signal error).

*^c^* The midpoint of transition, calculated from non-equilibrium unfolding/refolding experiments (Fig. 4).

*^d^* α-Value, indicating the ASA of the transition state of folding relative ASA of the native and unfolded state. *m_F_*, *m_U_*: linear dependence of ln(*k_F_*) and ln(*k_U_*) on GdnHCl concentration.

**Figure 5.**
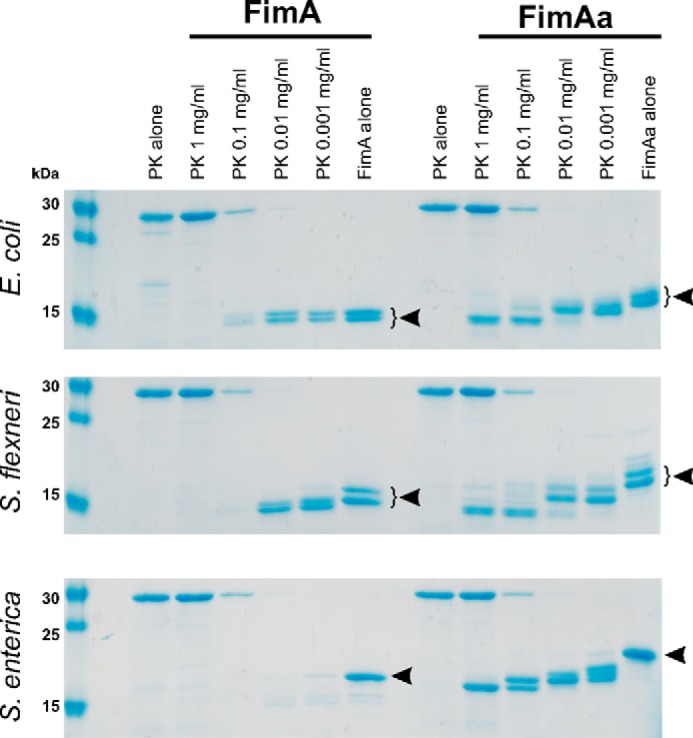
**Proteinase K resistance of the FimA monomers and the FimAa constructs.** FimA proteins (constant concentration of 20 μm) were incubated with different amounts of PK (0.001–1 mg/ml) for 30 min. Quenched, boiled, and reduced samples were run on an 18% polyacrylamide-SDS gel and are shown *above* for the three orthologues. Undigested proteins are labeled with *arrows*. All FimA monomers were completely digested at the highest PK concentrations. In contrast, a protease-resistant core with an apparent mass of ∼15 kDa was observed for all FimAa constructs, and PK only removed the ∼2–kDa segment corresponding to the N-terminal donor strand copy that was not incorporated into the FimAa-fold. FimA^ECO^, FimAa^ECO^, FimA^SHI^, and FimAa^SHI^ always ran as double bands on the SDS gel, despite the fact that all preparations showed a uniform mass corresponding to the full-length protein (Fig. S3). As the double bands did not disappear during partial digestion with PK and were reproducible for different preparations of the same protein, we can exclude that they resulted from proteolytic degradation prior to PK addition. We thus interpret the double bands as SDS-PAGE artifacts.

The incorporation of the C-terminal donor strand copy in all FimAa orthologues and their dramatically increased stability compared with the respective FimA monomers (Figs. 3 and 4) predicted that (i) the N-terminal donor strand copy in folded FimAa should be flexibly disordered and protease sensitive and (ii) that the folded core of FimAa should be more protease resistant than the less stable FimA monomers. Therefore, we tested all FimA/FimAa pairs for their resistance against proteinase K (PK). Fig. 5 shows that all FimA monomers were indeed completely degraded by PK, whereas only the N-terminal donor strand (∼2 kDa) was removed from the FimAa constructs, whereas the rest of the FimAa proteins remained PK resistant.

### The FimA donor strand does not contribute to the transition state of FimA folding

**Figure 6.**
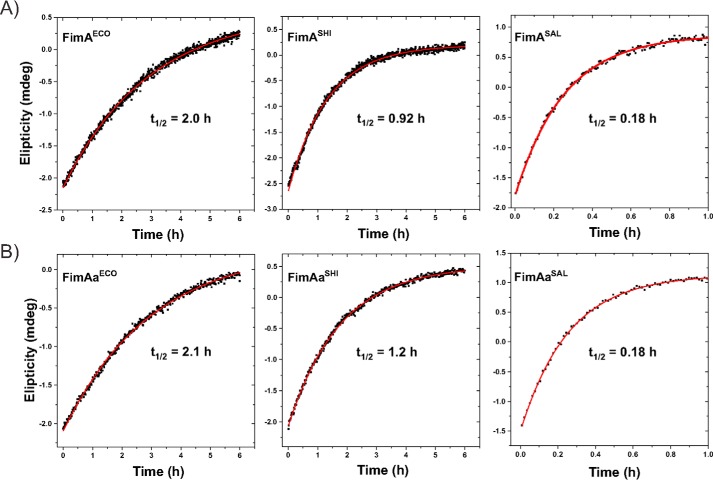
**Kinetics of spontaneous refolding of FimA monomers and the FimAa variants in 60 mm GdnHCl (pH 7.0) and 25 °C.** Refolding traces for FimA orthologues (*A*) and respective FimAa variants (*B*) are shown. Refolding was initiated by rapid dilution and monitored via the increase in the far-UV CD signal at 230 nm. The final protein concentration was 10 μm in all experiments. All kinetic data were consistent with first-order kinetics (*solid red lines*). The deduced half-lives are indicated for each protein.

Previous studies showed that pilus subunits show intrinsically slow kinetics of spontaneous folding, with half-lives between several minutes to 2 h (14, 17). Slow pilus subunit folding is likely caused by the high contact order of their complex β-sheet topology and catalyzed *in vivo* by the periplasmic chaperone FimC (14, 19, 26). To test whether the orientation of the incorporated donor strand influences the folding kinetics of FimA, we recorded the folding kinetics of all FimA and FimAa orthologues in the presence of identical residual GdnHCl concentrations of 60 mm via the increase in the far-UV CD signal at 230 nm. Fig. 6 and Table 3 show that nearly identical rate constants of folding were obtained for each FimA/FimAa pair at pH 7.0 and 25 °C, albeit the half-lives of folding varied between 0.18 (FimA^SAL^/FimAa^SAL^) and 2 h (FimA^ECO^/FimAa^ECO^) (Table 3). In addition, direct recording of the folding kinetics in 60 mm GdnHCl yielded essentially the same rate constants of folding as those predicted from the kinetic parameters deduced from the dependence of the ln(*k*_obs_) on GdnHCl concentration (Fig. 4*B*, Tables 2 and 3). These results indicate that most likely neither the N-terminal nor the C-terminal donor strand contributes to the stability of the transition state of FimA folding. If residues from the donor strands had contributed to transition state stability, we would have expected faster folding of the FimAa constructs compared with the respective FimA proteins due to the more stabilizing effect of the C terminally incorporated donor strand.

**Table 3:** **Comparison of the measured rate constants of folding in 60 mm GdnHCl of FimA monomers and the FimAa constructs with the rate constants predicted from the Chevron plots in Fig. 4*B***

FimA construct	Measured *k_F_* in 60 mm GdnHCl*^a^* (s^−1^)	Calculated *k_F_* in 60 mm GdnHCl*^b^* (s^−1^)	(*k*_calc_^*F*^)/(*k*_measured_^*F*^)	(*k_F_*(FimA))/(*k_F_*(FimAa))
FimA^ECO^	9.50 ± 0.08 × 10^−5^	NA*^c^*		1.03
FimAa^ECO^	9.26 ± 0.09 × 10^−5^	1.09 ± 0.28 × 10^−4^	1.18	
FimA^SHI^	2.08 ± 0.01 × 10^−4^	NA		1.32
FimAa^SHI^	1.57 ± 0.01 × 10^−4^	1.81 ± 0.28 × 10^−4^	1.15	
FimA^SAL^	1.05 ± 0.01 × 10^−3^	NA		0.97
FimAa^SAL^	1.08 ± 0.02 × 10^−3^	7.42 ± 1.09 × 10^−4^	0.69	

*^a^* Rate constants of folding at pH 7.0 and 25 °C in 60 mm GdnHCl, determined from the refolding experiments in Fig. 5.

*^b^* Rate constants of folding at pH 7.0 and 25 °C in 60 mm GdnHCl, calculated from the Chevron plots in Fig. 4*B* and the deduced values of *k_F_*^H2O^ and *m_F_* in Table 2.

*^c^* NA, not applicable.

## Discussion

In this article, we obtained strong evidence for a conserved, dual function of the type 1 pilus subunit FimA from enteroinvasive, Gram-negative bacteria that invade host cells via type 1 pilus-mediated adhesion. We showed that all three FimA orthologues investigated (FimA^ECO^, FimA^SHI^, and FimA^SAL^) were capable of folding to an alternative, self-complemented, monomeric form of only marginal thermodynamic stability that likely represents the FimA species that acts as an inhibitor of host cell apoptosis after internalization of the respective pathogen (21). Previous results indicated that FimA-mediated retardation of apoptosis is conferred by stabilization of the mitochondrial ion channel VDAC1-hexokinase complex against dissociation triggered by apoptotic stimuli (21). This mechanism raises the question of the origin of the FimA monomers in the cytosol of infected target cells. We consider the possibility that FimA monomers dissociate from assembled pilus rods very unlikely, because type 1 pilus rods are exceptionally stable and, for example, cannot even be dissociated by boiling, acidic pH or high GdnHCl concentrations at neutral pH (17, 24). Another possible source of FimA monomers could be periplasmic FimA molecules that remained assembly incompetent because they folded to monomers prior to binding to the assembly chaperone FimC. Although FimC rapidly binds unfolded FimA molecules newly translocated to the periplasm and accelerates FimA folding more than 10^4^-fold after binding, it is conceivable that a small fraction of secreted FimA molecules escape complex formation with FimC. For example, this could occur under conditions where the concentration of secreted FimA molecules is higher than the periplasmic concentration of FimC, so that a fraction of the FimA molecules would fold to assembly incompetent monomers independently of FimC.

Another notable result of our study is the striking stability difference between the FimA monomers and their respective FimAa counterparts. In the FimAa variants, analogous to FimA–FimA interactions in the assembled pilus rod, the C-terminal FimA donor strand was inserted in the antiparallel orientation into the FimA-fold. Although none of the three FimAa orthologues could be unfolded by incubation in 6 m GdnHCl for 1 day (Fig. 4*A*) and showed free energies of folding at pH 7.0 and 25 °C in the range of −60 to −70 kJ/mol (Table 2), all FimA orthologues were only marginally stable. Their free energies of folding between only −5.5 and −8.9 kJ/mol (Table 1) translate into 3–10% unfolded molecules at equilibrium under physiological conditions. Although it is generally difficult to predict thermodynamic stability differences from structural differences, the comparison of the FimA^ECO^ and FimAa^ECO^ structures revealed that a more extensive β-sheet interaction between the donor strand and the C-terminal F-strand of FimA (antiparallel in FimAa and parallel in FimA) might be a major factor that strongly increases the stability of FimAa relative to FimA (13).

The higher stability of the FimAa orthologues compared with the respective FimA monomers also became evident in our limited proteolysis experiments, which showed that only the FimA monomers could be completely degraded by PK (Fig. 5). In this context, it is interesting to note that during type 1-piliated bacterial infection, not only full-length FimA, but also shorter FimA peptide fragments were efficiently enriched on the surface of host mitochondria. Specifically, the 11-residue FimA fragment 50–60, corresponding to the entire FimA B-strand (Fig. S2), proved to be sufficient to target a FimA^60–70^–GFP fusion to mitochondria (21). Although a potential anti-apoptotic function of proteolytic FimA fragments has not yet been firmly established, our experiments demonstrated that FimA monomer degradation to peptides is strongly favored by their low intrinsic stability and these soluble monomers may provide a source of the anti-apoptotic peptide during infection.

## Materials and methods

### Primary structures of the FimA constructs used

FimA primary structures were from *E. coli* W3100 (K-12 WT strain), *S. enterica* subsp. *enterica* serovar Paratyphi A, strain A6043, and *S. flexneri* strain k-304. For expression in the *E. coli* cytoplasm, the natural signal peptides were deleted based on the signal peptidase cleavage site prediction with SignalP 4.1 (28). All FimAa variants were constructed as described (14, 29) by fusion of a (Gly)_6_-linker to the FimA C terminus, followed by a copy of the respective N-terminal donor strand.

### Protein expression and purification

FimA^ECO^ (159 amino acids, 15.8 kDa), FimA^SHI^ (162 amino acids, 16.4 kDa), FimA^SAL^ (164 amino acids, 16.6 kDa), FimAa^ECO^ (185 amino acids, 18.1 kDa), FimAa^SHI^ (189 amino acids, 18.9 kDa), and FimAa^SAL^ (190 amino acids, 19.0 kDa) were expressed as insoluble inclusion bodies in the *E. coli* cytoplasm under T7 promoter/lac operator control (expression vector pET11a) as described (14). Cells were grown in 2YT medium containing ampicillin (100 μg/ml) at 37 °C and harvested by centrifugation. Cells were suspended in 50 mm Tris-HCl (pH 8.0), 250 mm NaCl, and lysed with a microfluidizer at 12,000 PSI (five passages). Inclusion bodies were sedimented by centrifugation, solubilized with 6 m GdnHCl, 50 mm DTT, 50 mm Tris-HCl (pH 8.0), and applied to a 280-ml desalting column previously equilibrated with 6 m GdnHCl, 20 mm acetic acid-NaOH (pH 4.0). Eluted proteins were diluted to 5 μm and adjusted to pH 8.0 with 20 mm Tris-HCl. After addition of 0.1 μm CuCl_2_, the solution was incubated overnight at room temperature for Cu^2+^-catalyzed air oxidation of the single cysteine pair. The unfolded, disulfide-bonded proteins were concentrated via the cross-flow filtration (10 kDa cassettes) to ∼100 ml. Refolding was carried out by overnight dialysis of the protein solutions (50 μm) against 10 mm MOPS-NaOH (pH 7.0), 150 mm NaCl at 25 °C. Refolded proteins were applied to a GE Healthcare Superdex 75 10/300 GL column, and fractions corresponding to refolded protein were collected and concentrated by ultrafiltration (10 kDa cut-off filter). The yields of purified protein per liter of bacterial culture were about 40 mg for all FimA variants, and the identity of the purified proteins was confirmed by electrospray ionization-MS (Fig. S3).

### Protein concentrations

Protein concentrations were determined via the specific protein absorbance at 280 nm calculated with ProtParam (30): extinction coefficients were: FimA^ECO^, FimAa^ECO^: 2680 m^−1^ cm^−1^; FimA^SHI^, FimAa^SHI^: 4595 m^−1^ cm^−1^; FimA^SAL^, FimAa^SAL^: 4595 m^−1^ cm^−1^.

### GdnHCl-dependent unfolding and refolding transitions

Protein concentrations were kept constant at 10 μm in 10 mm MOPS-NaOH (pH 7.0) containing different GdnHCl concentrations, and incubated for 1–30 days at 25 °C. The final denaturant concentration in each sample was determined via the refractive index of the solution (31) after recording of the respective CD signal. Folding/unfolding was followed on Jasco J-715 spectropolarimeter via the far-UV CD signal change 230 nm, which was recorded for 30 s (1-s intervals) and averaged. The standard deviation of the recorded CD signals was less than 15% of the mean absolute ellipticity value for all samples.

Folding and refolding transitions of all FimA orthologues reached equilibrium after 1 day of incubation and were evaluated and normalized according to the two-state model as described (14). Unfolding and refolding transitions of FimAa orthologues were recorded after different incubation times and analyzed according to the theory of an unattained two-state equilibrium (23) using a combination of Equations 1–3,

(Eq. 1) $$k_{F}=k_{F}^{\text{H}2\text{O}}e^{m_{F}D}$$

(Eq. 2) $$k_{F}=k_{F}^{\text{H}2\text{O}}e^{m_{F}D}$$

(Eq. 3) $$f_{N}\left(t\right)=\frac{k_{F}}{k_{F}+k_{U}}+\left(f_{N}\left(0\right)-\frac{k_{F}}{k_{F}+k_{U}}\right)e^{-\left(k_{F}+k_{U}\right)t}$$

where *f_N_*(*t*) is the fraction of native molecules after incubation time *t*, *k_F_*^H2O^ and *k_U_*^H2O^ are the rate constants of folding and unfolding in the absence of denaturant, *m_F_* and *m_U_* are the linear dependences of ln(*k_U_*) and ln(*k_F_*) on GdnHCl concentration, and *D* is the GdnHCl concentration. The parameter *f_N_*(0) is zero for unfolding and one for refolding experiments. Equation 3 describes the kinetics of attainment of a two-state folding equilibrium, and Equations 1 and 2 describe the dependence of *k_F_* and *k_U_* on denaturant concentration, respectively. Replacement of *k_F_* and *k_U_* in Equation 3 by their denaturant dependences (Equations 2 and 3) yields the fraction of native molecules *f_N_*(*t*) as a function of *f_N_*(0), *D*, *k_F_*^H2O^, *k_U_*^H2O^, *m_F_*, *m_U_*, and incubation time *t*. As the folding equilibria of the FimAa constructs could not be attained, *k_U_* dominated over *k_F_* in the transition regions of unfolding, and *k_F_* dominated in the transitions regions of refolding. Therefore, *k_F_* was set to zero for global fitting of the three unfolding transitions recorded for each FimAa construct, and *k_U_* was set to zero for global fitting of the three refolding transitions. For normalization of the recorded CD signals, Equation 4 was used,

(Eq. 4) $$f_{N}=\frac{S-\left(S_{U}^{0}+m_{U}D\right)}{\left(S_{N}^{0}+m_{N}D\right)-\left(S_{U}^{0}+m_{U}D\right)}$$

where *S* is the measured CD signal, *S_N_*^0^ and *S_U_*^0^ are the signals of the folded and unfolded protein at zero denaturant, respectively, and *m_N_* and *m_U_* are the linear dependences of the signals of the folded and unfolded protein on *D*, respectively. For the refolding transitions of all FimAa constructs, *m_N_* was set to zero due to the absence of a pre-transition baseline.

V-plots (Fig. 4*B*) were deduced from the data points in the transition regions between 5 and 95% of folded molecules in Fig. 4*A*. From the fractions of folded molecules and the respective incubation times, the logarithm of the observed rate constants of folding/unfolding ln(*k*_obs_) was plotted against *D* and fitted according to Equation 5, with *k*_obs_ = *k_F_* + *k_U_*.

(Eq. 5) $${\text{lnk}}_{\text{obs}}=\ln\left(k_{F}^{\text{H}2\text{O}}\times e^{m_{F}\times \left[D\right]}+k_{U}^{\text{H}2\text{O}}\times e^{m_{U}\times \left[D\right]}\right)$$

The predicted equilibrium transitions of the FimAa constructs (*dotted lines* in Fig. 4*A*) were calculated from Equation 6,

(Eq. 6) $$f_{N}=\frac{e{-}^{\frac{\Delta G_{\text{H}2\text{O}}^{0}+m_{\text{eq}}\times D}{RT}}}{e{-}^{\frac{\Delta G_{\text{H}2\text{O}}^{0}+m_{\text{eq}}\times D}{RT}}+1}$$

where Δ*G*^0^_H2O_ is the free energy of folding at zero denaturant and *m*_eq_ is the cooperativity of folding (in J mol^−1^ m^−1^), which equals (*m_U_* − *m_F_*) × *RT*.

### Protein crystallization and X-ray data collection

Purified and concentrated FimA orthologues were crystallized using the sitting drop vapor diffusion method. Initial screens were performed at the Protein Crystallization Centre at the University of Zurich in MRC 96-well plates with 100 μl of reservoir and 100 nl of protein drop (FimA^ECO^ 23 mg/ml; FimA^SHI^ 23 mg/ml; and FimA^SAL^ 18 mg/ml, in 10 mm MOPS-NaOH (pH 7.0)). Refinement screens were performed in a 24-well plate with 1 ml of reservoir and 1 μl of protein drops of the same concentration as in the initial screens. Crystals were observed with the following precipitant solutions: FimA^ECO^: 0.1 m sodium malonate (pH 2.6), 46% ammonium sulfate, 4 °C; FimA^SHI^: 0.1 m sodium malonate (pH 3.0), 30% ammonium sulfate, 20 °C; FimA^SAL^: 0.34 m ammonium sulfate, 32.5% PEG 4k, 15% glycerol, 4 °C. Prior to data collection, crystals were soaked in 50% glycerol as a cryoprotectant. X-ray data were collected at the Swiss Light Source (beamlines X10SA and X6A). Diffraction data were processed and scaled using the XDS package (32).

### Structure solution and refinement

Structure determination was carried out by molecular replacement with Phaser (33), in the PHENIX software suite (34), using the FimA^ECO^ NMR structure (state 1) as a search model (PDB ID 2M5G). Iterative rounds of model building, refinement and validation were performed in COOT (27) and PHENIX, respectively. Structure analysis and visualization of the models were done in PyMOL (The PyMOL Molecular Graphics System, version 2.1, Schödinger LLC).

### Accession numbers

Atomic coordinates and structure factors for the reported FimA crystal structures have been deposited in the Protein Data Bank under the following accession codes: FimA^ECO^ (5NKT), FimA^SHI^ (5LP9), and FimA^SAL^ (6ERJ).

### Limited proteolysis of FimA and FimAa

Limited proteolysis of FimA and FimAa was performed at a constant protein concentration of 0.2 mg/ml in 20 mm Tris-HCl (pH 8.0), using Proteinase K concentrations of 1.0, 0.1, 0.01, or 0.001 mg/ml. The reactions were incubated at 37 °C for 30 min and stopped by addition of 5 mm phenylmethylsulfonyl fluoride and 5 mm EDTA. After addition of reducing SDS-PAGE loading buffer, samples were incubated for 5 min at 100 °C, separated on 18% SDS-polyacrylamide gels, and stained with Coomassie Instant Blue.

## Author contributions

D. S. Ż. and R. G. conceptualization; D. S. Ż., A. E. P., and G. C. data curation; D. S. Ż., A. E. P., G. C., and R. G. formal analysis; D. S. Ż., A. E. P., and R. G. validation; D. S. Ż. and A. E. P. investigation; D. S. x. and R. G. visualization; D. S. Ż., A. E. P., and R. G. methodology; D. S. Ż., A. E. P., G. C., and R. G. writing-original draft; D. S. Ż., G. C., and R. G. project administration; D. S. Ż., A. E. P., G. C., and R. G. writing-review and editing; G. C. and R. G. supervision; R. G. resources; R. G. funding acquisition.

## Supplementary Material

Supporting Information

## Footnotes

2 The abbreviations used are:

IG: immunoglobulin
VDAC: voltage-dependent anion channel
RMSD: root mean square deviation
GdnHCl: Guanidine HCl
ASA: accessible surface area
PK: proteinase K
PDB: Protein Data Bank.

## References

[1] Schilling J. D., Mulvey M. A., and Hultgren S. J. (2001) Structure and function of *Escherichia coli* type 1 Pili: new insight into the pathogenesis of urinary tract infections, J. Infect. Dis. 183, S36–S40 10.1086/318855 11171011

[2] Pizarro-Cerdá J., and Cossart P. (2006) Bacterial adhesion and entry into host cells. Cell 124, 715–727 10.1016/j.cell.2006.02.012 16497583

[3] Hospenthal M. K., Costa T. R. D., and Waksman G. (2017) A comprehensive guide to pilus biogenesis in Gram-negative bacteria. Nat. Rev. Microbiol. 15, 365–379 10.1038/nrmicro.2017.40 28496159

[4] Thanassi D. G., Bliska J. B., and Christie P. J. (2012) Surface organelles assembled by secretion systems of Gram-negative bacteria: diversity in structure and function. FEMS Microbiol. Rev. 36, 1046–1082 10.1111/j.1574-6976.2012.00342.x 22545799 PMC3421059

[5] Connell I., Agace W., Klemm P., Schembri M., Mărild S., and Svanborg C. (1996) Type 1 fimbrial expression enhances *Escherichia coli* virulence for the urinary tract. Proc. Natl. Acad. Sci. U.S.A. 93, 9827–9832 10.1073/pnas.93.18.9827 8790416 PMC38514

[6] O'Hanley P., Lark D., Falkow S., and Schoolnik G. (1985) Molecular basis of *Escherichia coli* colonization of the upper urinary tract in BALB/c mice; Gal-Gal pili immunization prevents *Escherichia coli* pyelonephritis in the BALB/c mouse model of human pyelonephritis. J. Clin. Invest. 75, 347–360 10.1172/JCI111707 2857730 PMC423490

[7] Sivick K. E., and Mobley H. L. (2010) Waging war against uropathogenic *Escherichia coli*: winning back the urinary tract. Infect. Immun. 78, 568–585 10.1128/IAI.01000-09 19917708 PMC2812207

[8] Dodson K. W., Pinkner J. S., Rose T., Magnusson G., Hultgren S. J., and Waksman G. (2001) Structural basis of the interaction of the pyelonephritic *E. coli* adhesin to its human kidney receptor. Cell 105, 733–743 10.1016/S0092-8674(01)00388-9 11440716

[9] Sauer M. M., Jakob R. P., Eras J., Baday S., Eriş D., Navarra G., Bernèche S., Ernst B., Maier T., Glockshuber R. (2016). Catch-bond mechanism of the bacterial adhesin FimH. Nat Commun. 7, 10738 10.1038/ncomms10738 26948702 PMC4786642

[10] Busch A., Phan G., and Waksman G. (2015) Molecular mechanism of bacterial type 1 and P pili assembly. Philos. Trans. R. Soc. A Math. Phys. Eng. Sci. 373, 20130153–20130153 10.1098/rsta.2013.0153 25624519

[11] Hospenthal M. K., Zyla D., Costa T. R. D., Redzij A., Giese C., Lillington J., Glockshuber R., and Waksman G. (2017) The cryoelectron microscopy structure of the type 1 chaperone-usher pilus rod. Structure 25, 1829–1838.e4 10.1016/j.str.2017.10.004 29129382 PMC5719983

[12] Spaulding C. N., Schreiber H. L. 4th, Zheng W., Dodson K. W., Hazen J. E., Conover M. S., Wang F., Svenmarker P., Luna-Rico A., Francetic O., Andersson M., Hultgren S., and Egelman E. H. (2018) Functional role of the type 1 pilus rod structure in mediating host-pathogen interactions. Elife 7, e31662 10.7554/eLife.31662 29345620 PMC5798934

[13] Walczak M. J., Puorger C., Glockshuber R., and Wider G. (2014) Intramolecular donor strand complementation in the *E. coli* type 1 pilus subunit fima explains the existence of fima monomers as off-pathway products of pilus assembly that inhibit host cell apoptosis. J. Mol. Biol. 426, 542–549 10.1016/j.jmb.2013.10.029 24184277

[14] Puorger C., Vetsch M., Wider G., and Glockshuber R. (2011) Structure, folding and stability of FimA, the main structural subunit of type 1 Pili from uropathogenic *Escherichia coli* strains. J. Mol. Biol. 412, 520–535 10.1016/j.jmb.2011.07.044 21816158

[15] Allen W. J., Phan G., and Waksman G. (2012) Pilus biogenesis at the outer membrane of Gram-negative bacterial pathogens. Curr. Opin. Struct. Biol. 22, 500–506 10.1016/j.sbi.2012.02.001 22402496

[16] Nishiyama M., Ishikawa T., Rechsteiner H., and Glockshuber R. (2008) Reconstitution of pilus assembly reveals a bacterial outer membrane catalyst. Science 320, 376–379 10.1126/science.1154994 18369105

[17] Puorger C., Eidam O., Capitani G., Erilov D., Grütter M. G., and Glockshuber R. (2008) Infinite kinetic stability against dissociation of supramolecular protein complexes through donor strand complementation. Structure 16, 631–642 10.1016/j.str.2008.01.013 18400183

[18] Vetsch M., Sebbel P., and Glockshuber R. (2002) Chaperone-independent folding of type 1 pilus domains. J. Mol. Biol. 322, 827–840 10.1016/S0022-2836(02)00845-8 12270717

[19] Vetsch M., Puorger C., Spirig T., Grauschopf U., Weber-Ban E. U., and Glockshuber R. (2004) Pilus chaperones represent a new type of protein-folding catalyst. Nature 431, 329–333 10.1038/nature02891 15372038

[20] Crespo M. D., Puorger C., Schärer M. A., Eidam O., Grütter M. G., Capitani G., and Glockshuber R. (2012) Quality control of disulfide bond formation in pilus subunits by the chaperone FimC. Nat. Chem. Biol. 8, 707–713 10.1038/nchembio.1019 22772153

[21] Sukumaran S. K., Fu N. Y., Tin C. B., Wan K. F., Lee S. S., and Yu V. C. (2010) A soluble form of the pilus protein FimA targets the VDAC-hexokinase complex at mitochondria to suppress host cell apoptosis. Mol. Cell 37, 768–783 10.1016/j.molcel.2010.02.015 20347420

[22] Kelley L. A., and Sutcliffe M. J. (1997) OLDERADO: On-line database of ensemble representatives and domains. Protein Sci. 6, 2628–2630 10.1002/pro.5560061215 9416612 PMC2143626

[23] Erilov D., Puorger C., and Glockshuber R. (2007) Quantitative analysis of nonequilibrium, denaturant-dependent protein folding transitions. J. Am. Chem. Soc. 129, 8938–8939 10.1021/ja0718927 17602628

[24] Eshdat Y., Silverblatt F. J., and Sharon N. (1981) Dissociation and reassembly of *Escherichia coli* type 1 pili. J. Bacteriol. 148, 308–314 6116696 10.1128/jb.148.1.308-314.1981 PMC216194

[25] Fersht A. (1998) Structure and mechanism in protein science: a guide to enzyme catalysis and protein folding. W. H. Freeman, New York, NY

[26] Vetsch M., Erilov D., Molière N., Nishiyama M., Ignatov O., and Glockshuber R. (2006) Mechanism of fibre assembly through the chaperone-usher pathway. EMBO Rep. 7, 734–738 10.1038/sj.embor.7400722 16767077 PMC1500831

[27] Emsley P., Lohkamp B., Scott W. G., and Cowtan K. (2010) Features and development of Coot. Acta Crystallogr. Sect. D Biol. Crystallogr. 66, 486–501 10.1107/S0907444910007493 20383002 PMC2852313

[28] Petersen T. N., Brunak S., G. von Heijne G., and Nielsen H. (2011) SignalP 4.0: discriminating signal peptides from transmembrane regions. Nat. Methods 8, 785–786 10.1038/nmeth.1701 21959131

[29] Barnhart M. M., Pinkner J. S., Soto G. E., Sauer F. G., Langermann S., Waksman G., Frieden C., and Hultgren S. J. (2000) PapD-like chaperones provide the missing information for folding of pilin proteins. Proc. Natl. Acad. Sci. U.S.A. 97, 7709–7714 10.1073/pnas.130183897 10859353 PMC16609

[30] Gasteiger E. et al (2005) The Proteomics Protocols Handbook. Humana Press, Totowa, New Jersey

[31] Nozaki Y. (1972) The preparation of guanidine hydrochloride. Methods Enzymol. 26, 43–50 10.1016/S0076-6879(72)26005-0 4680720

[32] Kabsch W. (2010) XDS Acta Crystallogr. Sect. D Biol. Crystallogr. 66, 125–132 10.1107/S0907444909047337 20124692 PMC2815665

[33] McCoy A. J., Grosse-Kunstleve R. W., Adams P. D., Winn M. D., Storoni L. C., and Read R. J. (2007) *Phaser* crystallographic software. J. Appl. Crystallogr. 40, 658–674 10.1107/S0021889807021206 19461840 PMC2483472

[34] Adams P. D. et al (2010) PHENIX: A comprehensive Python-based system for macromolecular structure solution. Acta Crystallogr. Sect. D Biol. Crystallogr. 66, 213–221 10.1107/S0907444909052925 20124702 PMC2815670

