# Supplementary material for: Alternative folding to a monomer or homopolymer is a common feature of the type 1 pilus subunit FimA from enteroinvasive bacteria
Source: J Biol Chem. 2019 May 24;294(27):10553–63. doi: 10.1074/jbc.RA119.008610 (PMC6615685; doi:10.1074/jbc.RA119.008610)
Supplement: Supporting Information [file supp_294_27_10553__index.html]

Alternative folding to a monomer or homopolymer is a common feature of the type 1 pilus subunit FimA from enteroinvasive bacteria — Alternative folding possibilities of the pilus subunit FimA — Alternative folding to a monomer or homopolymer is a common feature of the type 1 pilus subunit FimA from enteroinvasive bacteria — Alternative folding possibilities of the pilus subunit FimA — Supporting Information 

# Alternative folding to a monomer or homopolymer is a common feature of the type 1 pilus subunit FimA from enteroinvasive bacteria

## Supporting Information

- Supporting Information (to be published online) - Supporting Information in the PDF format
