# Supplementary material for: Alternative folding to a monomer or homopolymer is a common feature of the type 1 pilus subunit FimA from enteroinvasive bacteria
Source: J Biol Chem. 2019 May 24;294(27):10553–63. doi: 10.1074/jbc.RA119.008610 (PMC6615685; doi:10.1074/jbc.RA119.008610)
Supplement: Supporting Information [file supp_RA119.008610_144533_1_supp_334966_prql9m.pdf]

Alternative folding to a monomer or homopolymer is a common feature of the type 1 pilus subunit FimA from enteroinvasive bacteria

Dawid S. Żyła[1], Andrea E. Prota[2], Guido Capitani[2†], and Rudi Glockshuber[1*]

From the [1] Institute of Molecular Biology and Biophysics, ETH Zurich, Otto-Stern-Weg 5, CH-8093 Zurich, Switzerland; [2] Laboratory of Biomolecular Research, Division of Biology and Chemistry, Paul Scherrer Institute, Forschungsstrasse 111, CH-5232 Villigen PSI, Switzerland

[†]Deceased on 2 May 2017

Table of Contents

1. Figures S1-S3

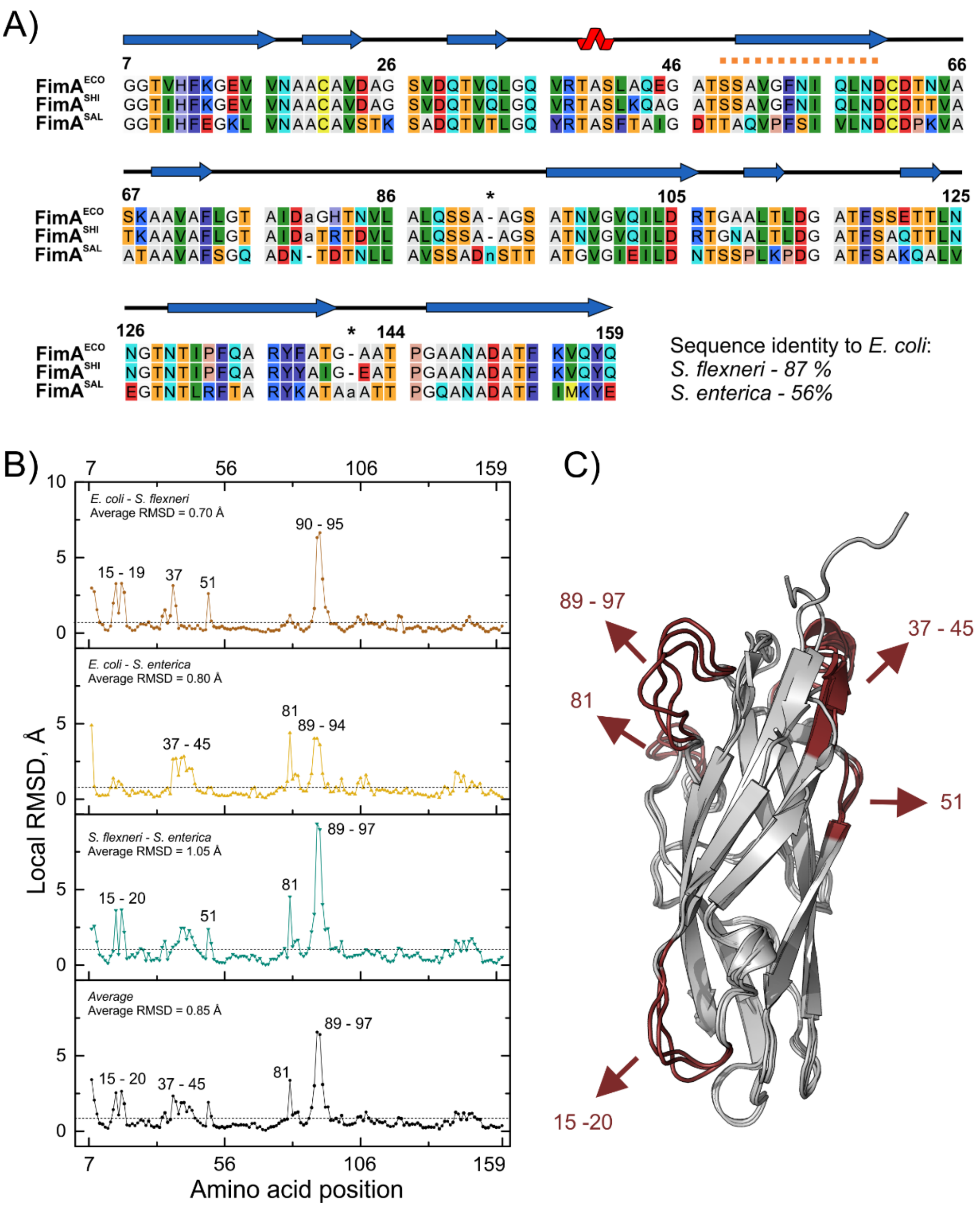

Figure S1: Pairwise structural alignment of the solved X-ray structures of the self-complemented monomers FimA^{ECO}, FimA^{SHI} and FimA^{SAL}.

A) Structure-based sequence alignment of FimA^{ECO}, FimA^{SHI} and FimA^{SAL} for the regions that were uniformly well resolved in the X-ray structures of all three. Secondary structures are indicated above the primary sequences. Amino acid numbering corresponds to mature *E. coli* FimA. Positions, where the sequences differ due to insertion of an additional residue (lower case letters), are marked with asterisks. The FimA fragment 50–60 that proved to be sufficient for targeting a GFP fusion protein to mitochondria is indicated as an orange dotted line [21].

B) The plot of the pairwise, local RMSD values against residue number.

C) Superposition of all solved structures. Polypeptide segments with the highest structural variability are highlighted in red.

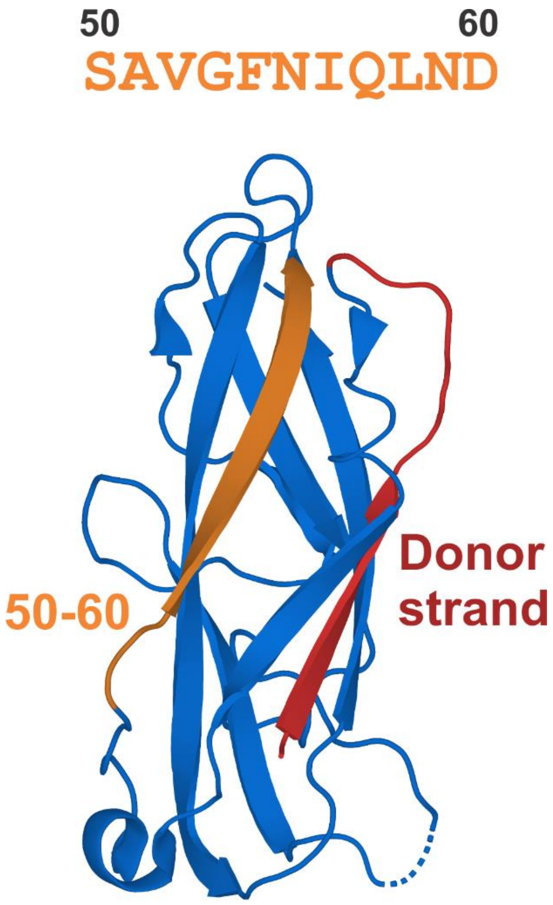

Figure S2: Localisation of the FimA anti-apoptotic peptide segment 50–60 in the context of the solved X-ray structure of FimA^{ECO} (orange). The segment 50–60, which corresponds to the FimA β-strand (amino acid sequence indicated) proved to be sufficient to specifically target GFP to mitochondria when fused to the GFP N-terminus [21]. The self-complementing donor strand is coloured in red.

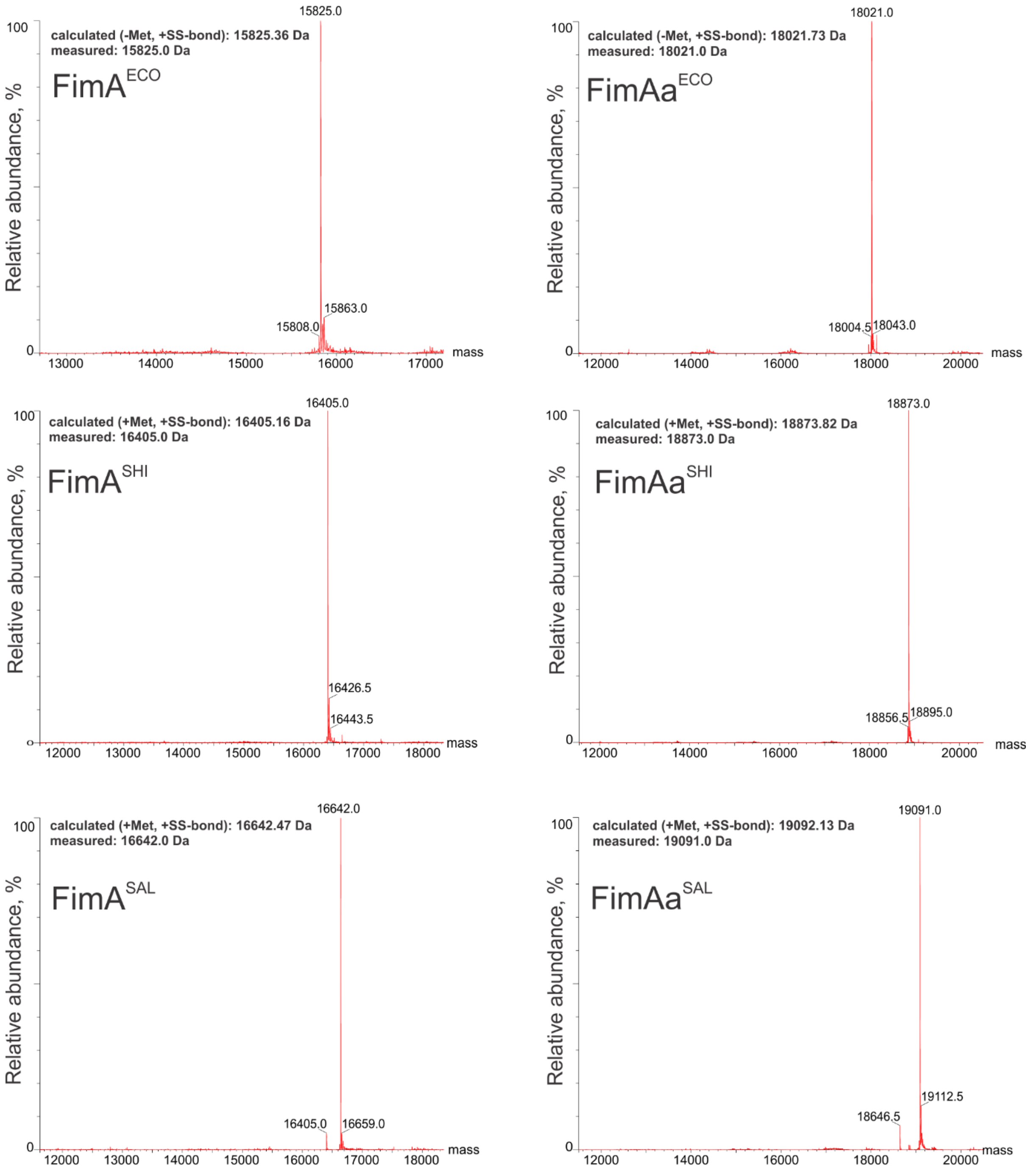

Figure S3: ESI-MS spectra of FimA and FimAa orthologues. All measured masses are consistent with calculated masses. All proteins contain a single disulfide bond. For FimA^{SHI} and FimA^{SAL} variants, the N-terminal methionine was not cleaved.
